# Supplementary material for: Association of Cesarean Delivery with Trajectories of Growth and Body Composition in Preschool Children
Source: Nutrients. 2022 Apr 26;14(9):1806. doi: 10.3390/nu14091806 (PMC9103341; doi:10.3390/nu14091806)
Supplement: Supplementary file 1 [file nutrients-14-01806-s001.zip › Table S1.pdf]

Table S1. Mean zBMI growth rates (SD units per month) during each growth period from the unadjusted and adjusted analyses [a]

Growth period	Unadjusted model			Adjusted model [b]		
	**Mean (SE) Growth rate**	**95% CI**	***P***	**Mean (SE) Growth rate**	**95% CI**	***P***
0~6, month	0.029 (0.005)	0.019, 0.039	<0.001	0.029 (0.005)	0.019, 0.039	<0.001
6~36, month	–0.016 (0.001)	–0.017, –0.015	<0.001	–0.016 (0.001)	–0.017, –0.015	<0.001
>36, month	0.0004 (0.001)	–0.001, 0.002	0.404	0.0005 (0.001)	–0.001, 0.002	0.356

[a] Piecewise linear mixed models were used to model mean zBMI growth rates. [b] Adjusted for maternal age, maternal education, annual family income, prepregnancy BMI, gestational weight gain, gravidity, parity, gestational age, child sex, birthweight, breastfeeding duration, and parent-reported dietary intake of children. Abbreviations: zBMI, BMI z score.
