# Supplementary material for: Association of Cesarean Delivery with Trajectories of Growth and Body Composition in Preschool Children
Source: Nutrients. 2022 Apr 26;14(9):1806. doi: 10.3390/nu14091806 (PMC9103341; doi:10.3390/nu14091806)
Supplement: Supplementary file 1 [file nutrients-14-01806-s001.zip › Table S2.pdf]

Table S2. Adjusted mean differences of anthropometric and body composition measures for cesarean delivery compared with vaginal delivery (reference), stratified by child birthweight or sex

	β Coefficient	95%CI	*P*	*P* for interaction test
**FMI, kg/m^2**				
Birthweight (g)				
<2500	0.461	(0.011, 0.910)	0.045	0.306
2500~3999	0.087	(0.013, 0.161)	0.021	
≥4000	0.210	(–0.126, 0.547)	0.221	
Sex				
Male	0.133	(0.035, 0.230)	0.008	0.159
Female	0.058	(–0.047, 0.162)	0.282	
**FFMI, kg/m^2**				
Birthweight (g)				
<2500	0.241	(–0.003, 0.484)	0.053	0.499
2500~3999	0.011	(–0.035, 0.057)	0.641	
≥4000	0.128	(–0.039, 0.294)	0.134	
Sex				
Male	–0.011	(–0.072, 0.049)	0.714	0.347
Female	0.051	(–0.013, 0.115)	0.118	
**FM%**				
Birthweight (g)				
<2500	2.039	(–0.408, 4.487)	0.102	0.258
2500~3999	0.384	(0.006, 0.762)	0.047	
≥4000	0.522	(–1.000, 2.043)	0.501	
Sex				
Male	0.596	(0.124, 1.068)	0.013	0.154
Female	0.185	(–0.322, 0.693)	0.474	
**zBMI**				
Birthweight (g)				
<2500	–3.045	(–8.195, 2.106)	0.247	0.354
2500~3999	0.059	(–0.004, 0.121)	0.065	
≥4000	0.144	(–0.108, 0.395)	0.264	
Sex				
Male	0.077	(–0.011, 0.166)	0.087	0.370
Female	0.057	(–0.024, 0.139)	0.167	

Abbreviations: FMI, fat mass index; FFMI, fat-free mass index; FM%, percentage of body fat; zBMI, BMI z score.
