# Supplementary figures and images for: Association of Cesarean Delivery with Trajectories of Growth and Body Composition in Preschool Children
Source: Nutrients. 2022 Apr 26;14(9):1806. doi: 10.3390/nu14091806 (PMC9103341; doi:10.3390/nu14091806)

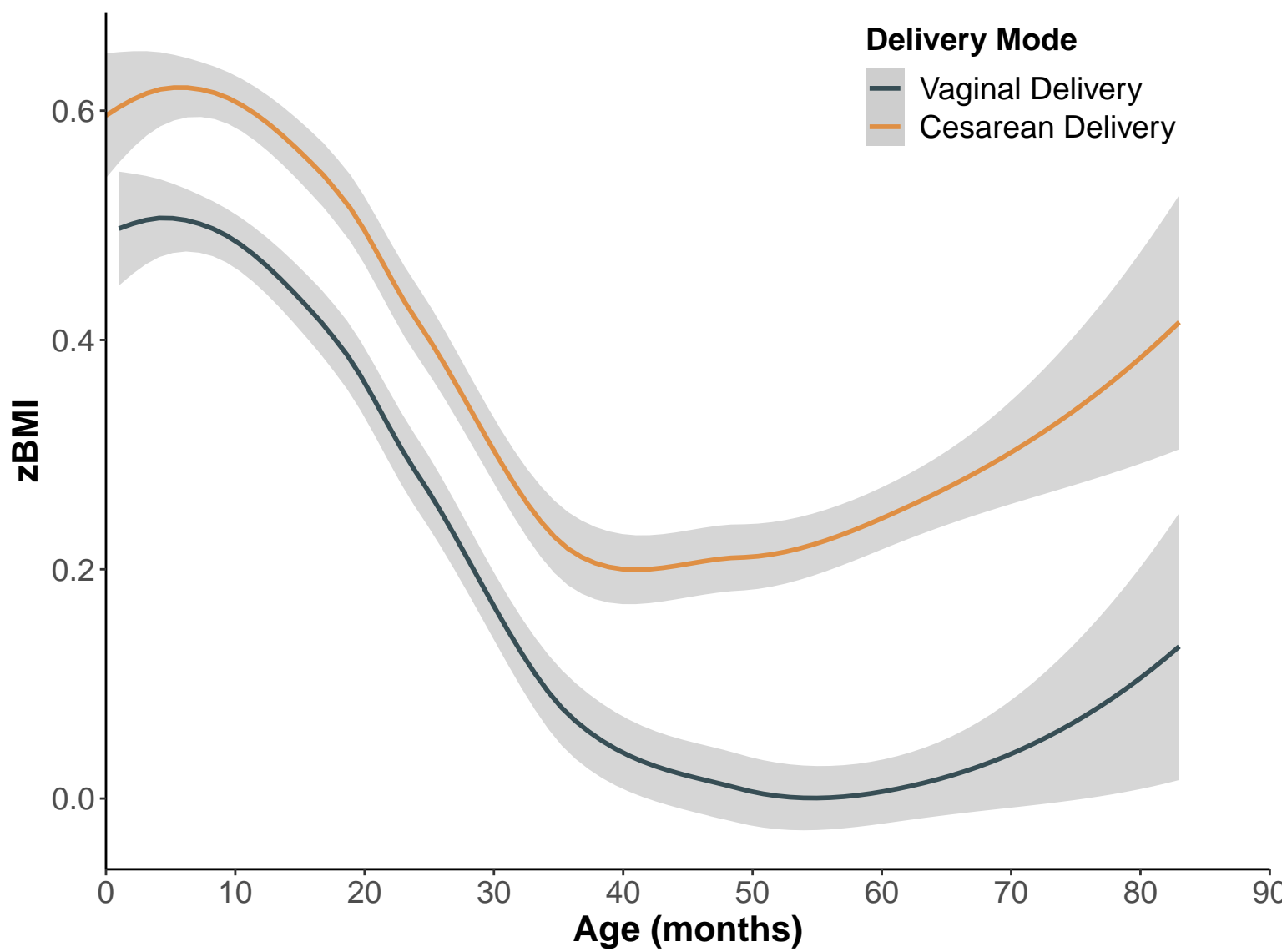

Supplement: Supplementary file 1 [file nutrients-14-01806-s001.zip › Figure S1.pdf]
